# Unlocking the Microbial Symphony: The Interplay of Human Microbiota in Cancer Immunotherapy Response

**DOI:** 10.3390/cancers17050813

**Published:** 2025-02-26

**Authors:** Jessica Chacon, Farah Faizuddin, Jack C. McKee, Aadil Sheikh, Victor M. Vasquez, Shrikanth S. Gadad, Ghislaine Mayer, Sharon Siby, Molly McCabe, Subramanian Dhandayuthapani

**Affiliations:** 1 Foster School of Medicine, Texas Tech University Health Sciences Center El Paso, El Paso, TX 79905, USA; fafaizud@ttuhsc.edu (F.F.); jack.c.mckee@ttuhsc.edu (J.C.M.); aadishei@ttuhsc.edu (A.S.); shrikanth.gadad@ttuhsc.edu (S.S.G.); damayer@ttuhsc.edu (G.M.); sharon.siby@ttuhsc.edu (S.S.); momccabe@ttuhsc.edu (M.M.); s.dhandayuthapani@ttuhsc.edu (S.D.); 2 L. Frederick Francis Graduate School of Biomedical Sciences, Texas Tech University Health Sciences Center El Paso, El Paso, TX 79905, USA; 3 Center of Emphasis in Cancer, Department of Molecular and Translational Medicine, Texas Tech University Health Sciences Center El Paso, El Paso, TX 79905, USA; 4 Center of Emphasis in Infectious Diseases, Department of Molecular and Translational Medicine, Texas Tech University Health Sciences Center El Paso, El Paso, TX 79905, USA

**Keywords:** microbiota, immunotherapy, cancer, microbiome

## Abstract

Introduction: The emergence of cancer immunotherapy has revolutionized cancer treatment, offering remarkable outcomes for patients across various malignancies. However, the heterogeneous response to immunotherapy underscores the necessity of understanding additional factors influencing treatment efficacy. Among these factors, the human microbiota has garnered significant attention for its potential role in modulating immune response. Body: This review explores the intricate relationship between the human microbiota and cancer immunotherapy, highlighting recent advances and potential mechanisms underlying microbial influence on treatment outcomes. Conclusion: Insights into the microbiome’s impact on immunotherapy response not only deepen our understanding of cancer pathogenesis but also hold promise for personalized therapeutic strategies aimed at optimizing patient outcomes.

## Simple Summary

Cancer immunotherapy has transformed how we treat cancer, leading to significant improvements for many patients. However, not all individuals respond the same way to these treatments, which suggests that other factors influence their effectiveness. One such factor is the collection of bacteria and other microorganisms that naturally live in and on the human body, known as the microbiome. Scientists have begun to explore how these microorganisms interact with the immune system and whether they can affect how well immunotherapy works. This review discusses the connection between the microbiome and cancer immunotherapy, examining recent discoveries and possible ways these microorganisms influence treatment success. Understanding this relationship could lead to more personalized approaches to cancer treatment, improving outcomes for patients by tailoring therapies based on their microbiome.

## 1. Introduction

Cancer immunotherapy, particularly checkpoint blockade, has emerged as a transformative approach in oncology by harnessing the body’s immune system to target and destroy cancer cells. Checkpoint inhibitors, specifically those targeting Cytotoxic T-lymphocyte associated protein-4 (CTLA-4) and Programmed cell death protein-1 (PD-1/PD-L1) pathways, work by blocking the inhibitory signals that cancer cells use to evade immune detection [1]. Normally, CTLA-4 and PD-1 are immune checkpoint proteins that help regulate immune responses and prevent autoimmunity by inhibiting T-cell activation and proliferation when bound to their ligands [2]. However, cancer cells can exploit these checkpoints to escape immune surveillance. By administering antibodies that block CTLA-4 or PD-1/PD-L1, checkpoint inhibitors effectively “release the brakes” on T-cells, enhancing their ability to recognize and attack tumor cells. This immune activation has shown remarkable success in treating various cancers, including melanoma, non-small cell lung cancer, and renal cell carcinoma [2].

With the growing emphasis on immunotherapy as a promising cancer treatment, it has become essential to understand the factors influencing patient responses to therapy in order to minimize immune-related adverse events (irAEs) and optimize treatment efficacy [3]. One emerging factor in influencing responses to immunotherapy is the microbiome. The microbiome refers to the collection of microorganisms, including bacteria, viruses, fungi, and their genetic material, that live in and on the human body, playing a crucial role in maintaining health and influencing various biological processes [4]. Specifically, the gut microbiota has demonstrated a strong influence on host physiology in maintaining homeostasis under normal conditions [5]. Changes to microbial community structure and function, or dysbiosis, can have negative health effects on the host [6]. Factors such as disease, dietary, and environmental changes can shift the proportions of the microbial community, which can lead to chronic inflammation, influence the development and prognosis of diseases such as cancer, and impact the response to treatment [7]. The microbiome, particularly the gut microbiome, considerably has been shown to influence chemotherapy by affecting its effectiveness and toxicity since it can directly affect how patients process and respond to the chemotherapy drugs [8]. Although many studies have reported on the usefulness of nanotherapeutic agents to reverse the dysbiosis of inflammatory bowel disease and dental caries, the microbiome may also be able to influence both targeted and nanotherapies [9,10].

Though most of the research has been focused on the gut microbiome, there is an increasing recognition of the role of the intratumor microbiome in modulating the local tumor microenvironment (TME) [11]. Distinct intestinal and intratumor microbial community signatures are associated with carcinogenesis, prognosis, and treatment outcomes across various cancers [12]. Recent studies have demonstrated that the enrichment of the TME or intestinal tract with certain microbes can improve responses to immunotherapy, and conversely, dysbiotic communities can decrease treatment effectiveness [13]. The relationship between the microbiome and immunotherapy response underscores the potential of manipulating the microbiota, either through diet, probiotics, or fecal microbiota transplantation (FMT), to enhance the effectiveness of checkpoint blockade therapy [13]. This review article will explore the relationship between the human microbiota and cancer immunotherapy, focusing on recent advances in understanding how microbial factors influence treatment efficacy and the potential for personalized therapeutic strategies to enhance patient outcomes.

## 2. Optimizing Patient Selection and Mechanistic Insights in Cancer Immunotherapy

Selecting patients for immunotherapy at times requires specific biomarkers and genetic factors that can predict treatment success. One of the most critical biomarkers is PD-L1 expression in the TME. High PD-L1 expression levels are often associated with improved responses to PD-1/PD-L1 inhibitors, making it a key consideration in patient selection [14].

### 2.1. Role of Tumor Mutational Burden, Microsatellite Instability, and Mismatch Repair Deficiency

Another important factor is tumor mutational burden (TMB), which measures the number of mutations within a tumor’s DNA. Tumors with high TMB produce more neoantigens—unique proteins that make the tumor more recognizable to activated T-cells, thus enhancing the immune response [15]. Genetic factors also play a significant role, particularly the status of microsatellite instability-high (MSI-H) or deficient mismatch repair (dMMR) [16]. These genetic alterations have been strongly linked to better outcomes with immune checkpoint inhibitors [17]. By incorporating these biomarkers into clinical decision-making, healthcare providers can take a more personalized approach, ensuring that patients who are most likely to benefit from immunotherapy receive priority in treatment plans [18,19].

### 2.2. Immune Checkpoint Inhibitors: Mechanisms and Targets

**Figure 1:**
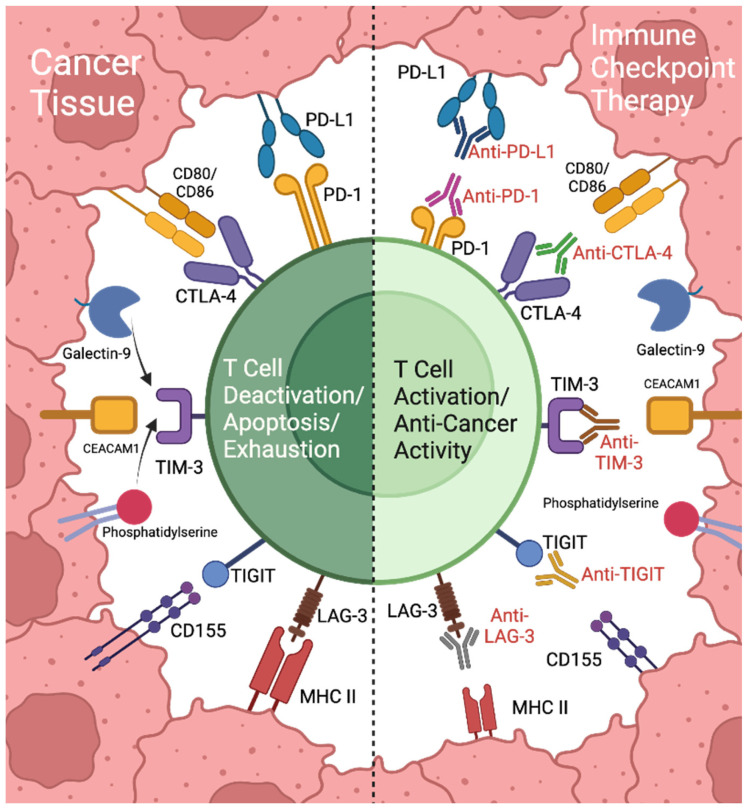
Immune checkpoints and their role in immunosuppression and immune checkpoint inhibitor therapy. Immune checkpoints CTLA-4, PD-1, LAG-3, TIM-3, and TIGIT bind to their ligands on cancer cells, contributing to decreased T cell activity and immunosuppression. Immune checkpoint inhibitor therapy utilizes antibodies targeting these proteins and their ligands, disrupting their interactions and promoting anti-cancer immune activity. Created with BioRender.com.

Immune checkpoint inhibitors (ICIs) work by disrupting inhibitory pathways that limit the immune system’s ability to recognize and destroy cancer cells, targeting specific immune checkpoints like CTLA-4, PD-1, PD-L1, lymphocyte activation gene 3 (LAG-3), T-cell immunoglobulin and mucin domain-3 (TIM-3), and T-cell immunoglobulin and ITIM (TIGIT) (Figure 1). Each of these checkpoints helps regulate immune responses to prevent overactivation and autoimmunity by maintaining a balance between immune priming and activation [20]. CTLA-4, for example, is highly expressed in regulatory T cells (Tregs), which play a critical role in immune tolerance by suppressing excessive immune responses. CTLA-4 competes with the costimulatory receptor Cluster of Differentiation 28 (CD28) for binding to B7 molecules on antigen-presenting cells, reducing the priming of new T-cells [21]. In contrast, PD-1/PD-L1 interactions primarily affect activated T-cells within the TME, dampening their response and allowing cancer cells to evade detection [22]. By using checkpoint inhibitors to block these receptors or their ligands, the immune system can overcome suppression by “releasing the brakes” on the immune system, promoting a more robust priming and activation of T-cells, enabling sustained immune responses against tumor cells. This mechanism underscores the success of checkpoint inhibitors in enhancing anti-tumor immunity, although variability in individual immune systems and tumor biology can impact patient responses.

LAG-3, TIM-3, and TIGIT are additional immune checkpoints that contribute to immune suppression, particularly within the TME, and they each have unique mechanisms of action. LAG-3 binds to MHC class II molecules on antigen-presenting cells, which inhibits T-cell proliferation and cytokine production, leading to reduced immune activation [23]. LAG-3 is often co-expressed with PD-1 on T-cells, and its inhibition has been shown to further rejuvenate T-cells and enhance their anti-tumor activity [24]. TIM-3 binds to several ligands, including Galectin-9, phosphatidylserine, and Carcinoembryonic antigen-related cell adhesion molecules (CEACAM1), resulting in T-cell exhaustion by interfering with T-cell receptor (TCR) signaling, reducing cytokine production and promoting T-cell apoptosis [25]. TIM-3 is often expressed on Tregs and exhausted effector T-cells, and its role in regulating immune tolerance makes it a key checkpoint in sustaining chronic immune suppression in the TME [26]. TIGIT binds to CD155 on antigen-presenting cells and tumor cells, competing with the activating receptor 226 CD226 [27]. When TIGIT binds to CD155, it sends inhibitory signals through Immunoreceptor tyrosine-based inhibitory motifs (ITIM) in its cytoplasmic tail, which dampens T-cell and natural killer (NK) cell activity and decreases cytokine release [28]. TIGIT expression is associated with T-cell exhaustion, and blocking it can restore T-cell functionality and increase anti-tumor immune responses [28].

The expression of these checkpoints contributes to immune exhaustion and evasion of the immune system. By inhibiting these pathways, T-cell activation and cytokine production are enhanced, improving T-cell persistence and efficacy against cancer cells. This multi-targeted approach is especially valuable, as blocking multiple checkpoints simultaneously has been shown to further amplify anti-tumor immunity and overcome resistance in cases where single checkpoint inhibition alone may be insufficient [29]. Tumor-infiltrating lymphocytes (TILs) are gaining significant attention in cancer immunotherapy due to their recent FDA approval and pivotal role in directly combating tumors. TIL are immune cells that infiltrate the TME and include diverse subsets such as cytotoxic CD8+ T-cells, helper CD4+ T-cells, and NK cells [30], among others. The presence of high levels of TILs in tumors is often linked to better patient outcomes and an enhanced response to ICIs [31]. TIL therapy works by isolating TILs from a patient’s tumor, expanding them ex vivo, and reinfusing the billions of TIL back into the patient to amplify the body’s antitumor immune response [32]. TIL therapy has shown remarkable success in treating melanoma, achieving durable responses, even in cases of treatment-resistant disease [33]. By focusing on TILs and factors that influence TIL efficacy, such as the TME, researchers aim to refine immunotherapy by leveraging the tumor-specific activity of these immune cells, offering new hope for improving outcomes in cancer patients.

### 2.3. Tumor-Infiltrating Lymphocytes in Cancer Immunotherapy

TIL therapy represents the immune system’s effort to recognize and attack cancer cells within the tumor. However, the activity of TILs is often curtailed by immune checkpoint pathways, such as PD-1/PD-L1 and CTLA-4, which tumors exploit to evade immune destruction. ICI therapies have revolutionized cancer treatment by blocking these pathways, restoring T-cell activity, and promoting tumor regression in many patients. Despite these advances, the effectiveness of ICI therapies is significantly influenced by the TME—a complex ecosystem of immune cells, stromal components, and signaling molecules. The TME can create a suppressive environment that impairs TIL function and limits ICI efficacy. Exploring the dynamic interplay between TILs, immune checkpoint pathways, and the TME is crucial for developing strategies to overcome resistance and improve immunotherapeutic outcomes.

### 2.4. Chimeric Antigen Receptor T-Cell Therapy and Non-Solid Tumors

Most immune therapies like ICIs and TILs are indicated for solid tumors. Chimeric Antigen Receptor T-cell (CAR-T) therapies primarily treat hematologic conditions: B-ALL, lymphomas, and multiple myeloma [34]. With these, the patient’s own T-cells are genetically engineered to express receptors for tumor antigens. The molecular elements of the receptors can be optimized to suit the characteristics of certain cancers. For example, because B-cell lymphomas and leukemias abundantly express CD-19, antigen receptors for the CD-19 surface marker are among the most commonly used. New and upcoming generations of CAR-T therapy are optimized with intra-cellular costimulatory domains. Adjustments like these not only more closely resemble the signaling patterns utilized by natural T-cells to activate and proliferate but also enhance the function and longevity of therapeutic cell lines. The 4th generation of CARs, known as TRUCKs, even include an IL-12-secreting domain in addition to costimulatory and intracellular activating domains. Currently 6 CAR-T therapies have been approved by the FDA to treat the aforementioned malignancies and are the standard of care [34].

### 2.5. Patient Factors and Considerations Influencing Immunotherapy

Advancing age significantly impacts the gut microbiome and thus contributes to the outcome of immunotherapy treatments. As we age, decreasing appetite, poor dentition or nutrient intake, and other factors causing malnourishment disrupt the gut microbiome leading to increases of pro-inflammatory species such as *Enterobacteriaceae* and *Proteobacteria* [35]. This imbalance leads to inflammation, reduced synthesis of vitamins, malabsorption, and anabolic resistance [36,37,38], contributing to a dysbiosis in the body that can impair the efficacy of immune checkpoint inhibitors and other common therapies. Furthermore, the composition of the gut microbiota is influenced by dietary habits throughout a patient’s lifetime. Fiber-rich, plant-based diets, for example, enhance gut microbiome diversity [39], increasing the population of beneficial species that produce metabolic and immune regulating compounds, decreasing oxidative stress and chronic inflammation [40]. Conversely, diets high in saturated fats or highly processed foods, characteristic of many diets in the United States, contribute to dysbiosis and inflammation as a result of the disruption in the microbiome. Studies have demonstrated that personalized dietary intervention in combination with probiotics and prebiotics have potential to attenuate these disruptions to the microbiome [41,42] and thus positively influence immunotherapy treatment response.

## 3. The Tumor Microenvironment

The TME plays a pivotal role in shaping the effectiveness of immunotherapies. This complex environment consists of cellular and non-cellular elements, including immune cells, stromal cells, blood vessels, the extracellular matrix, and soluble factors like cytokines and growth factors [43]. Unfortunately, the TME often works against the immune system, creating conditions that suppress T-cell activation and function. Key mechanisms of immunosuppression include the recruitment of regulatory T-cells (Tregs) and myeloid-derived suppressor cells (MDSCs), the secretion of inhibitory cytokines like TGF-β and IL-10, and metabolic changes such as hypoxia and elevated lactate levels [44]. Together, these factors establish a hostile environment for effective antitumor immunity. Targeting the TME to overcome these barriers is a promising strategy to enhance immunotherapy [45].

### 3.1. Non-Biochemical Challenges: Cellular Interactions in the Tumor Microenvironment

**Figure 2:**
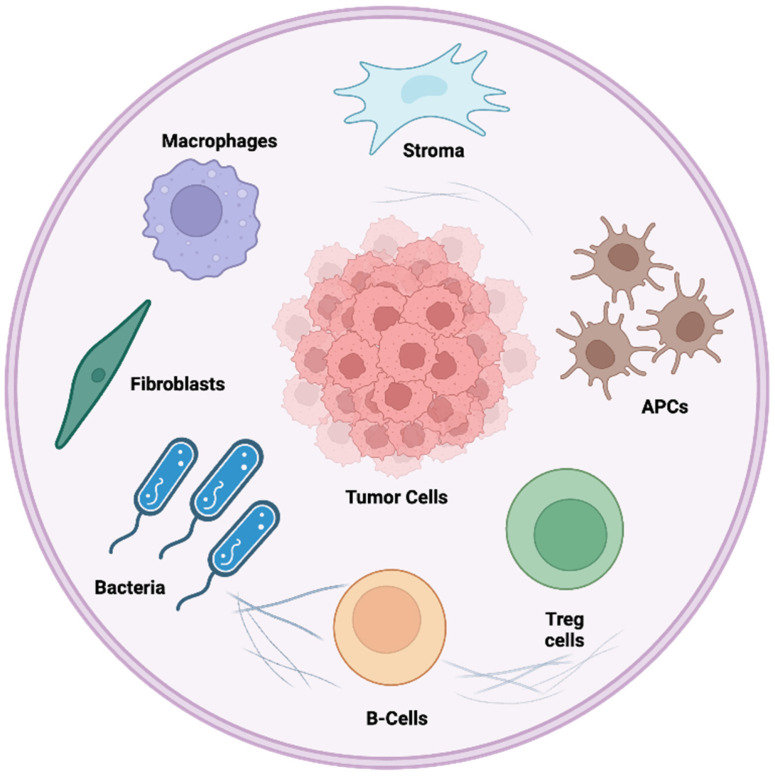
The tumor microenvironment contains diverse elements that influence cancer progression and outcomes. Different populations of immune cells, fibroblasts, stromal components, and microbes work in tandem to maintain niches in the tumor that can potentially influence responses to treatment. Created with BioRender.com.

As previously mentioned, the TME represents a dynamic and multifaceted ecosystem encompassing cellular, molecular, and structural elements that interact with tumor cells. A critical component of the TME is cells such as tumor-associated macrophages (TAMs), dendritic cells (DCs), NK cells, MDSCs, and TILs (Figure 2). These cells can exert anti-tumor effects through immune surveillance, but when functionally impaired, may facilitate tumor growth and metastasis [45,46]. Tumor resident DCs form the majority of the antigen-presenting cells (APCs) in the TME; they phagocytose and process cancer antigens for presentation to effector T cells, which can drive immunosurveillance within the TME [47]. Other components of the TME, such as Tregs, fibroblasts, and cancer cells, secrete various cytokines, chemokines, and metabolites that can determine whether the DCs maintain pro- or anti-cancer activity, making these factors a promising target to enhance immunotherapy responses. TAMs can promote cancer by secreting cytokines that reduce CD8+ T-cell infiltration, leading to an immunosuppressive TME that supports cancer progression [48]. NK cells are critical effector cells of the innate immune system that utilize several mechanisms to recognize, target, and eliminate target cells while sparing healthy cells. The primary mechanism of action relies on the recognition of MHC-I deficient cells and the release of perforins and granzymes to promote cell lysis in a process called “missing self-recognition” [49,50]. This mechanism serves to complement T-cell mediated immunity. However, immunosuppressive factors in the TME, such as anti-inflammatory cytokines, M2 macrophages, and Tregs, mitigate their activity and reduce the elimination of cancer tissue [49,50]. T cells form a significant portion of the TIL population within the TME, including populations of Tregs. While high levels of TILs in the TME are associated with better prognosis and treatment outcomes, persistent activation of these cells in the tumor leads to a loss of effector functions and the expression of inhibitory receptors, leading to T-cell exhaustion [48]. While Tregs normally promote immunotolerance and prevent autoimmunity, they are a prominent fixture within the TME that attenuates anti-cancer responses [50]. These cell populations, in conjunction with diverse stromal components within the TME, determine cancer progression, treatment response, and recurrence.

In addition to immune cell interactions, the microbiome plays a crucial role in shaping the immune landscape of the TME. Certain gut microbial species, such as *Bifidobacterium* and *Akkermansia muciniphila*, have been linked to enhanced immunotherapy responses by promoting dendritic cell activation and T-cell infiltration [51]. Conversely, pathogenic bacteria such as *Fusobacterium nucleatum* within tumors can promote immune evasion, suppressing anti-tumor immunity. Microbial metabolites, including short-chain fatty acids (SCFAs), influence immune cell differentiation and function, further modulating the balance between pro- and anti-tumor immune responses [52]. Additionally, the microbiome modulates inflammatory pathways through interactions with Toll-like receptors (TLRs) and the production of immune-modulating metabolites such as polyamines and tryptophan derivatives, which can either enhance or suppress anti-tumor immunity depending on the microbial composition [53]. Certain bacteria can also influence the differentiation of macrophages into either pro-inflammatory (M1) or immunosuppressive (M2) phenotypes, further shaping the immune environment of the tumor [54].

### 3.2. Non-Biochemical Challenges: Stromal Components Within the Tumor Microenvironment

Stromal components, including cancer-associated fibroblasts (CAFs), the extracellular matrix (ECM), and vascular structures, provide essential biochemical and structural support. However, they also act as physical barriers that hinder drug delivery and create niches that shield tumor cells from therapeutic interventions [46,55]. The ECM is particularly influential in malignant transformation by undergoing remodeling involving alterations in proteins, glycoproteins, and proteoglycans as well as modulating extracellular signaling pathways [56].

### 3.3. Biochemical Challenges: Hypoxia and Metabolic Reprogramming in the Tumor Microenvironment

Hypoxia is another hallmark of the TME, characterized by the activation of hypoxia-inducible factors (HIFs). This metabolic reprogramming drives tumor cells to rely on glycolysis for energy and suppresses anti-tumor immune responses mediated by CD8+ T cells, B cells, and NK cells, while promoting the expansion of immunosuppressive populations such as MDSCs and Tregs [57]. Hypoxia also compromises the delivery and efficacy of treatments like chemotherapy (CTX) and immunotherapy [57]. The metabolic changes in the TME provide a niche for microbial colonization in different body compartments, enabling the presence of bacteria, fungi, and other microorganisms that can influence cancer and patient physiology.

## 4. The Microbiome’s Role in Cancer Biology: From Gut Microbiota to Tumor Microenvironments

The microbiome, particularly the gut microbiota, is increasingly recognized as a key player in cancer biology. It influences cancer progression through mechanisms such as chronic inflammation, the disruption of epithelial barriers, and the production of metabolites that promote tumorigenesis [58]. Dysbiosis accelerates these processes as pathogenic microbes outcompete symbiotic species, diminishing their beneficial effects on the host [59]. Altered microbial communities in the gut and tumor microenvironment have been observed in cancer patients compared to healthy individuals, with significant implications for treatment response and prognosis [60].

### 4.1. The Microbiome in Homeostasis and Disease

Microbiome colonization begins at birth, with maternal bacteria transferred to the infant through delivery and breastfeeding. During vaginal delivery, infants are exposed to maternal vaginal, fecal, and skin microbiota, which play a critical role in early immune development. Common bacterial genera in this process include *Lactobacillus*, *Streptococcus*, *Bacteroides*, and *Bifidobacterium* [61,62]. In contrast, infants delivered via Cesarean section (C-section) are primarily colonized by skin-associated and hospital-environment microbes, leading to decreased proportions of *Bifidobacterium*, *Lactobacillus*, and *Bacteroides* and an increased representation of *Streptococcus*, *Staphylococcus*, *Propionibacterium*, and *Clostridium difficile* [61,62]. While the differences between the two cohorts even out several months after delivery, there are still unique community compositions such as higher *Clostridium* species representations in C-section infants and higher *Bacteroides* in vaginally delivered babies [62,63].

The introduction of solid food further shifts the microbiome, favoring species from the *Firmicutes* and *Bacteroidetes* phyla. This microbial composition remains relatively stable throughout life but adapts to intrinsic physiological and external environmental changes [62,63].

Disruptions to an established microbiome, including shifts in microbial composition or metabolite production, can result in dysbiosis—an imbalance linked to various diseases. Dysbiosis can enable pathogenic species to outcompete commensals, reducing their beneficial functions and contributing to conditions such as cardiovascular diseases, cancer, and respiratory disorders. Imbalances in gut microbiota can drive inflammation and elevate the risk of chronic conditions like diabetes, obesity, heart disease, irritable bowel syndrome (IBS), asthma, depression, and anxiety [64]. Notably, individuals with obesity exhibit reduced microbial diversity compared to healthy individuals, and germ-free mice transplanted with microbiota from obese individuals gain more weight than controls, highlighting the microbiome’s role in host metabolism [65,66,67].

The lung microbiota has also been implicated in chronic respiratory diseases [64]. Individuals with asthma exhibit increased bacterial load and diversity, with a higher abundance of *Proteobacteria* and lower levels of *Bacteroidetes* and *Firmicutes*. Furthermore, fungal communities in asthma patients differ from those in individuals without asthma [64,68]. These findings underscore how microbial dysbiosis across different tissue niches can contribute to pathological states and negatively impact host health.

### 4.2. The INTRA-Tumor Microbiome

In addition to the gut microbiome, the microbes that colonize tumor niches and constitute the intratumor microbiome provide an important aspect of the TME that have wide-ranging local cellular effects, much like the gut microbiome’s systematic influence on host physiology [69]. In gastric cancer (GC), *Helicobacter pylori* is a key pathogen driving sustained inflammation, DNA damage, and metabolic alterations [70]. Its effects include the generation of carcinogenic N-nitroso compounds, such as N-nitrosamines and nitric oxide, which are linked to both gastric and colorectal cancer development [71,72].

### 4.3. Microbial Metabolites in Cancer Biology

Microbial metabolites, such as secondary bile acids produced by *Clostridium* species, play a significant role in cancer biology. These metabolites suppress anti-tumor immune responses, including the downregulation of CD8+ T cell activity [73]. Secondary bile acids, like deoxycholic acid (DCA), promote colorectal cancer (CRC) by inducing oxidative DNA damage, mitochondrial stress, and chromosomal instability [74]. In breast cancer, alterations in microbial composition, such as the increased abundance of *Methylobacterium radiotolerans* in malignant tissues, are associated with cancer progression, metastasis, and resistance to therapies [75]. Some bacterial populations can deactivate chemotherapeutic agents or enhance immune evasion, thereby complicating treatment strategies [76].

### 4.4. Microbial Modulation of the Tumor Immune Microenvironment

Bacteria also influence the tumor immune microenvironment (TIME) by modulating immune cell activity or facilitating immune evasion through antigen mimicry and interactions with pattern recognition receptors (PRRs). For example, *H. pylori* evades immune recognition by altering its surface molecules,, such as lipopolysaccharides (LPS) and flagellin, to inhibit detection by toll-like receptors (TLRs) and c-type lectin-mediated signaling [50]. Flagellin’s dual activation of TLR5 and the NLRC4 inflammasome is a mechanism that downregulates TLR5-mediated antibody responses, aiding bacterial persistence [77]. Further, *H. pylori* impairs adaptive immune responses by interfering with antigen presentation, further promoting tumor immune evasion [50]. It is also worth mentioning that there has been a growing interest surrounding small-molecule inhibitors featuring six-membered aromatic nitrogen heterocycles. More specifically, recent literature has gone to show that these compounds not only facilitate antitumor processes but also contribute to modulating immune responses—potentially enhancing therapeutic effectiveness [78].

Tissue niches that were originally believed to be sterile and devoid of microbes, such as the liver and the lower respiratory tract, have been shown to host a complex microbiota [50]. Dysbiosis within these communities has been implicated in the exacerbation of chronic conditions and can induce cancer-promoting chronic inflammation [50]. Mutations in certain genes that encode immunity-related functions are associated with inflammation in the TME and potentially hold a dynamic relationship with tissue-resident microbiota. The functional loss of p53 results in the induction of multiple inflammatory-related pathways such as the upregulation of TNF-α, and NF-κB-associated cytokines and chemokines [79]. Additionally, it has been demonstrated that the microbiome in p53-mutant mice promotes cell proliferation through WNT signaling and opposes p53-mediated WNT suppression. Further analysis revealed that the production of microbial polyphenols can interfere with p53 function and lead to WNT hyperactivation [80]. *KRAS* mutations are frequently observed in cancers that are closely associated with chronic inflammation, such as pancreatic ductal adenocarcinoma (PDAC), CRC, and lung adenocarcinoma [81]. Patients with *KRAS* mutant cancers are shown to have microbiomes with a higher abundance of *Roseburia*, *Parabacteroides*, *Staphylococcus*, and *Staphylococcaceae* than patients with non-mutant *KRAS* cancers, suggesting that interplay between microbial and mutational profiles influences cancer development [82].

Together, the components of the TME, including immune cells, stroma, microbiomes, and genetic profiles, maintain a dynamic, significant influence on cancer pathophysiology, including initiation and progression. They can also influence patient responses to treatment modalities, including CTX, radiation (RT), and immunotherapy.

## 5. The Microbiome and Cancer Treatment

Current options for cancer treatment include traditional methods such as CTX, RT, and surgery, along with emerging technologies such as ICIs and targeted therapy [83]. There is a growing body of evidence showing that the microbiota of the gut and other sites plays an essential role in modulating the response and toxicity of treatments through a number of proposed mechanisms [84].

### 5.1. Chemotherapy and the Microbiome

**Figure 3:**
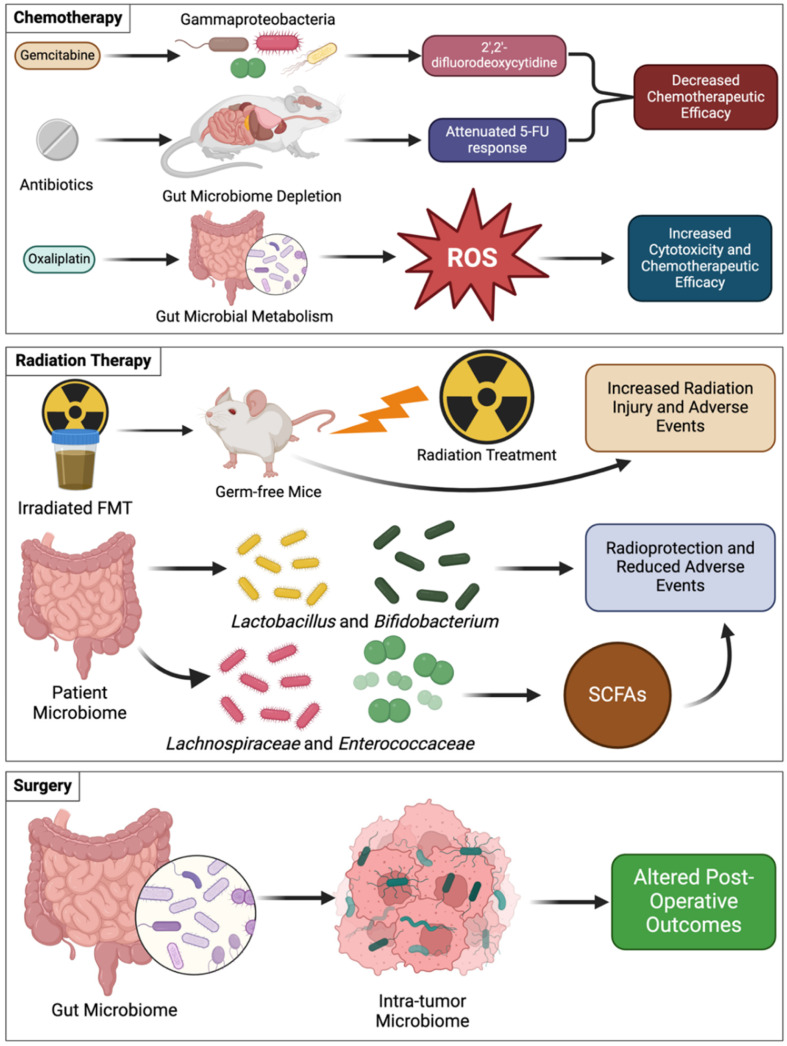
The microbiome influences outcomes in classical cancer treatment modalities. Gammaproteobacteria can convert gemcitabine into an inactive metabolite, reducing its efficacy in PDAC. Additionally, antibiotic depletion of the microbiome can reduce the efficacy of 5-FU and oxaliplatin. Oxaliplatin can work synergistically with resident gut microbiota that generate ROS that enhance the cytotoxicity of the drug. FMT studies have demonstrated that microbiomes that have been subjected to radiation can lead to poor prognosis and adverse events to radiotherapy. Conversely, it has been found that patient microbiomes that are enriched with *Lactobacillus*, *Bifidobacterium*, *Lachnospiraceae*, and *Enterococcaceae* have improved responses to radiotherapy and fewer adverse events, possibly due to the production of short-chain fatty acids (SCFAs) that promote anti-inflammatory pathways. Analyses of patient microbiomes have demonstrated a dynamic equilibrium between the gut and intra-tumor microbiomes that influence post-operative outcomes. Created with BioRender.com.

CTX is a critical component of cancer care, often considered for first-line use, and its utility depends on patient age, tumor location, size, stage, and genetic markers [50]. Commonly used CTX agents include cyclophosphamide, oxaliplatin, 5-fluorouracil (5-FU), and gemcitabine, all of which can be metabolized by the microbiome with situation-dependent effects (Figure 3) [50,85]. 5-FU is a fluorinated uracil analog that acts primarily by inhibiting the synthesis of thymidylate [36,86]. Studies have shown that the antibiotic depletion of the gut microbiota in the mouse colorectal cancer models can attenuate the effects of 5-FU and oxaliplatin [87]. The removal of the microbiome greatly reduces the efficacy of 5-FU, while conversely, supplementing the microbiome with probiotic genera such as *Lactobacillus* and *Bifidobacterium* did not improve the anti-cancer effects of the drug when compared to 5-FU treatment alone [87]. Likewise, gemcitabine, another nucleoside analog (2′,2′-difluorodeoxycytidine), is used to treat a variety of cancers arising from breast, testicular, ovarian, bladder, and pancreatic tissues [88]. A study demonstrated that gemcitabine can be metabolized into its inactive form (2′,2′-difluorodeoxyuridine) by the bacterial enzyme cytidine deaminase, commonly found in species belonging to the Gammaproteobacteria class [89,90]. Gemcitabine is often used to treat PDAC. To determine if bacteria are present in PDAC samples, 16S sequencing was performed, and 76% of the samples were found to have bacterial DNA, with the most common species identified belonging to the Gammaproteobacteria class, suggesting that intratumor bacteria are an integral part of the TME and modulate resistance to gemcitabine in PDAC [89]. Another common chemotherapeutic agent is oxaliplatin. It is a platinum-based alkylating agent that cross-links DNA and prevents cell division [91]. The antibiotic depletion of the microbiome decreases the effectiveness of oxaliplatin, similarly to 5-FU [92]. The commensal genera of the intestinal tract were shown to generate reactive oxygen species (ROS) that activate pro-inflammatory responses, inducing oxaliplatin cytotoxicity [92]. These findings demonstrate that CTX is influenced by the microbiome in a context-dependent manner where microbes metabolize the chemotherapeutic agents to either enhance or dampen their cytotoxic properties. In addition to microbial influences on chemotherapy metabolism, novel studies have demonstrated the utilization of microrobots for tumor drug delivery. The microrobots implement a sophisticated drug encapsulation and targeted path editing, possibly altering the interaction between chemotherapeutic agents and the microbiome and ultimately treatment outcomes [93].

### 5.2. Radiation Therapy and the Microbiome

RT has been included in cancer treatments for decades and advancements have enabled improved efficacy [94]. Yet, varying degrees of tumor sensitivity and radio-resistance development during treatment still present a major challenge to RT outcomes. The off-target effects of RT extend to the microbiome as radiation-induced changes to community structure promote negative health outcomes [95]. The analysis of radiation-treated murine fecal matter showed an increase in beta diversity compared to controls and an over-representation of opportunistic pathogens such as *Proteobacteria* and *Bacteroides* [96]. FMT studies demonstrate that inoculating germ-free mice with an irradiated microbiome increases their susceptibility to RT-induced injury. Additionally, the treatment of the mice with IL-1 receptor antagonists mitigated radiation-induced damage and inflammation [96]. Other findings have demonstrated that patients who were given *Lactobacillus* and *Bifidobacterium* prior to RT had fewer irAEs relating to treatment. Research has shown that microbiomes containing *Lachnospiraceae* and *Enterococcaceae* can provide radio-protective effects to patients and prevent the development of irAEs through the production of SCFAs, which inhibit inflammatory pathways [96]. The investigation of the intratumor microbiome has shown that the colonization of the TME with *Lactobacillus iners* can promote radio-resistance and decrease recurrence-free survival by up-regulating the formation of lactic acid and promoting metabolic reprogramming of the tumor [97].

### 5.3. Surgery and the Microbiome

While the role of the microbiome in surgery remains underappreciated, emerging studies are establishing an association of patient gut microbiome with post-operative outcomes in cases of colorectal cancers and PDAC [98]. In cases of PDAC, it was observed that the gut microbiome can modulate the intratumor microbiome composition and that patients with higher tumor alpha diversity had better long-term survival outcomes [99]. Another study demonstrated that tumor colonization by butyrate-producing bacteria and low alpha diversity was associated with recurrence in lung cancer [100]. Together, the findings suggest that the interaction of the microbiome with RT and surgery can influence carcinogenesis and health outcomes. However, more research is required to fully elucidate the mechanisms that govern these interactions.

### 5.4. Non-Bacterial Components of the Microbiome

While the role of the microbiome in health and disease is becoming more appreciated, the contribution of the viral, protozoal, and fungal components of the microbiome remains underappreciated. Fungal dysbiosis enhances responses to radiation, and the treatment of orthotopic lung and skin cancer mouse models with antifungals showed increased tumor burden reduction and increased survival following RT [101]. Further analysis showed that tumors that were colonized with *Candida albicans* and *Saccharomyces* were more likely to be radioresistant compared to samples treated with fluconazole [101]. *C. tropicalis* is closely related to colorectal carcinogenesis and can promote chemoresistance to oxaliplatin through lactate production and the inhibition of mismatch repair proteins [102,103]. The virome’s role in carcinogenesis is still subject to investigation because no direct or causal association has been established [90]. While several viruses including Epstein Barr virus (EBV), human papillomavirus (HPV), hepatitis B virus (HBV), hepatitis C virus (HCV), and human T-cell lymphotropic virus type 1 (HTLV-1), have been classified as cancer promoters, little has been investigated in the interaction between the virome and cancer treatment [90,104]. Certain bacteriophages are associated with microbial dysbiosis in colorectal cancer by targeting commensal microbes, enabling the proliferation of pathogenic species that promote cancer progression and may contribute to treatment resistance and poor outcomes [105]. Protozoa are less commonly studied in this context; however, they may still contribute to cancer outcomes. An association between cancer and *Tyrpanosomoa cruzi* has been observed due to the development of Chagas disease [90]. Patients with megacolon and megaesophagus due to chronic *T. cruzi* infection were observed to have increased mutations in *p53* and aneuploidies in chromosomes 7, 11, and 17, which may increase the risk of cancer development [106]. These genetic changes may also contribute to responses in cancer treatment, though much work is still required to establish an association between protozoans and cancer treatments. By considering fungi, viruses, and protozoa alongside bacteria, researchers can gain a more comprehensive understanding of the microbiome’s role in cancer prognosis, treatment response, and relapse risk.

### 5.5. The Microbiome and Tumor-Infiltrating Lymphocyte Therapy

The microbiome plays a pivotal role in shaping the success of TIL therapy by modulating the host immune system and the TME. The gut microbiota, in particular, influences systemic immune responses through the production of metabolites like short-chain fatty acids and bile acids, which can enhance or suppress T cell activity. A healthy, symbiotic microbiome supports the activation and persistence of TILs by fostering a pro-inflammatory environment conducive to anti-tumor immunity [107]. Conversely, dysbiosis or the overgrowth of pathogenic microbes can impair TIL efficacy by promoting immune evasion, chronic inflammation, and immunosuppression within the TME [108]. Emerging evidence suggests that microbiome composition may influence TIL therapy outcomes, with specific microbial species enhancing T cell activation and infiltration [109]. Understanding these microbiome–TIL interactions offers opportunities to optimize TIL therapy, potentially through microbiome modulation strategies such as probiotics, dietary interventions, or fecal microbiota transplantation, and to improve treatment efficacy and patient outcomes.

The microbiome plays a complicated role in cancer progression, treatment responses, and outcomes. The numerous species of bacteria, fungi, viruses, and protozoa and their interactions with conventional cancer treatment options provide an opportunity to use the microbiome as a marker for monitoring and predicting prognosis and treatment outcomes. As research into cancer therapeutic development advances, particularly with the rise of immunotherapy and personalized medicine options, it is evident that the gut and intratumor microbiomes need to be considered to maximize treatment efficacy.

## 6. The Microbiome and the Response to Immunotherapy

The microbiome plays a critical role in shaping the immune system and influencing the response to immunotherapy. Specific gut microbiota have been linked to an improved efficacy of ICIs, CAR-T, hematopoietic stem cell transplantation (HSCT), and more, likely by modulating immune activation and inflammation. Understanding the interaction between the microbiome and immunotherapy may lead to novel strategies to enhance treatment outcomes.

### 6.1. Influence of the Microbiome on ICI Therapy Efficacy

**Figure 4:**
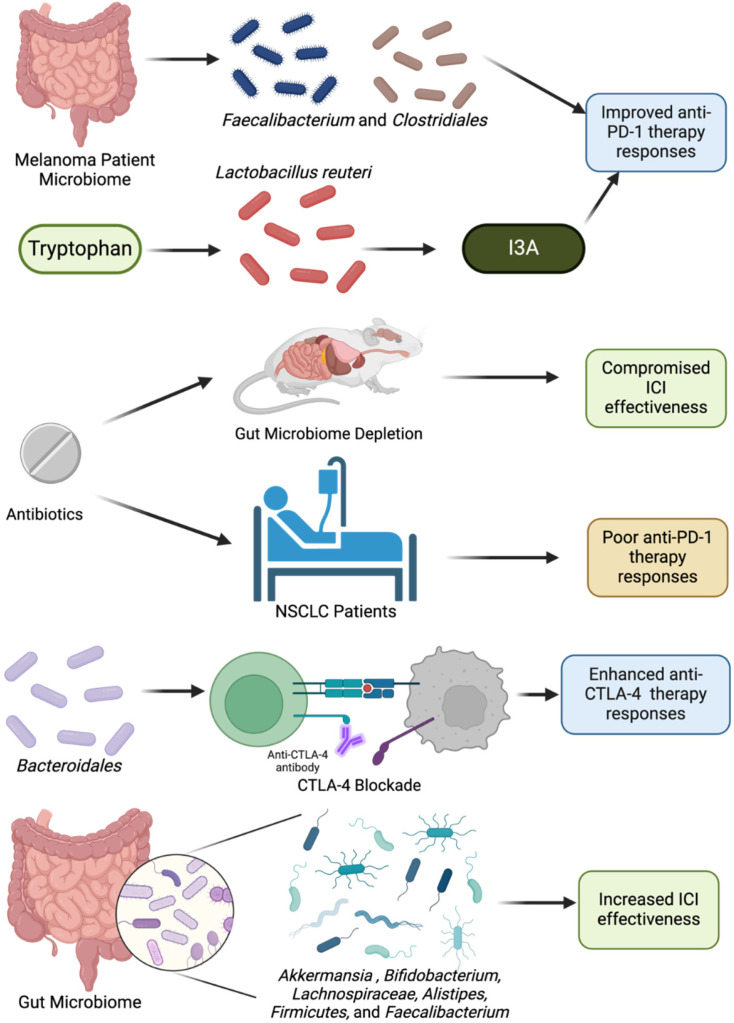
The microbiome influences ICI therapy outcomes. Fecal matter analysis of patients with melanoma demonstrated that responders to anti-PD-1 treatment had a microbiota that was enriched in *Faecalibacterium* and *Clostridiales*. Additionally, intratumor microbiomes that contained *Lactobacillus reuteri* showed improved responses to anti-PD-L1 therapy due to the metabolism of tryptophan into I3C. Antibiotic treatment of mice and patients is associated with diminished responses to ICI treatment, while certain genera such as *Alistipes*, *Bifidobacterium*, and *Bacteroidales* are associated with increased ICI effectiveness. Created with BioRender.com.

Since solid tumors comprise a majority of the cancer burden in the population, ICI therapy for solid tumors is a consistent subject of microbiome cancer research, and thus here is given special attention. ICI therapy has seen wide success in cancer treatment, yet increasing evidence shows that intestinal and intratumor microbiota can influence treatment efficacy and responsiveness (Figure 4) [110,111]. For instance, a groundbreaking study by Gopalakrishnan and colleagues found that melanoma patients with higher levels of beneficial bacteria like *Faecalibacterium* and *Clostridiales* experienced better outcomes when treated with anti–PD-1 therapy. This evidence highlights how the gut microbiome’s composition can directly affect the efficacy of cancer immunotherapies. The antibiotic depletion of the intestinal microbiome in mouse models can compromise ICI efficacy [89]. CTLA-4 blockade relies on *Bacteroidales* species to promote successful anti-cancer T-cell responses [112,113]. Additionally, treating non-small cell lung cancer patients with antibiotics prior to receiving anti-PD1/PD-L1 therapies resulted in lower progression-free survival and overall survival [62]. Such outcomes can be attributed to decreased species richness and altered community structure, including the depletion of commensal bacteria genera such as *Ruminococcaceae* [114]. It has been found that patients who respond well to ICI therapy tend to have microbiomes with an increased abundance of *Akkermansia muciniphila*, *Bifidobacterium*, *Lachnospiraceae*, *Alistipes*, *Firmicutes*, *Bacteroides,* and *Faecalibacterium* [62,63,64,65,66,67]. These bacteria are thought to enhance immune cell infiltration in the TME and promote an anti-tumor immune response by increasing the production of metabolites and cytokines that favor T-cell activation [89]. Moreover, the microbiome’s influence is not limited to treatment response—it also affects immune-related side effects. Certain microbial profiles have been linked to a lower risk of colitis in patients undergoing CTLA-4 blockade, suggesting the potential of the microbiome to reduce some adverse effects of immunotherapy. Microbial metabolites, such as SCFAs, are generated through the fermentation of dietary fiber, a process influenced by individual diet and microbiome composition. SCFAs like butyrate and propionate not only enhance immune regulation but also correlate with better long-term responses to anti-PD-1 therapy, underscoring the importance of dietary interventions in optimizing treatment outcomes [115,116,117].

### 6.2. Microbiome Modulating Factors and Non-Solid Tumors

With regards to the gut microbiome, current evidence suggests that there may be just as many profound, far-reaching effects on cancer progression and response to therapies as there are for solid tumors. According to a 2020 study by Vicente-Dueñas et al., mice that were predisposed to developing B-cell ALL, when given antibiotics developed leukemia versus mice not given antibiotics. Not only that, but mice with the leukemia predisposition did not have as robust of a gut biome at baseline [118].

Patients with adequate gut biome diversity and bacterial populations do not suffer as many deleterious side effects from immunotherapies including CAR-T and Hematopoietic Stem Cell Transplants (HSCT), which are commonly used to treat hematologic cancers, or from radiation and ICI, which can also be utilized in battling these diseases. It is important to note that they do so without reducing the efficacy of the treatment. The bacterial populations associated with this benefit in CAR-T and HSCT have significant overlap with those that do so for ICIs and other immunotherapies. Specifically with regard to CAR-T, several active metabolites of bacteria including the short-chain fatty acids butyrate and pentanoate appear to increase the activity and serum population of CAR cytotoxic T-cells [118].

Another treatment modality used often in tumors of the blood and lymph, HSCT, is affected by the gut microbiome. Higher levels of certain bacteria including *Eubacteriaceae*, *Bacteroides,* and *Blautia* were found to be associated with an increased efficacy of HSCT, and a low diversity of gut organisms was associated with an increased incidence of Graft versus Host Disease (GvHD). FMT, prebiotic supplementation, and probiotic supplementation can be helpful to improve patient outcomes. There is, in fact, more verified knowledge of specific bacterial populations and byproducts that help prevent GvHD following HSCTs than for other cancer treatments. In the near future, we foresee having a set of recommendations for clinical settings for patients receiving HSCT therapy, aimed at increasing the health of the butyrate-producing bacterial colonies [119].

### 6.3. The Role of Intratumor Microbes in Immunotherapy for Solid Tumors

A majority of the studies conducted so far focus on gut microbes, yet there are few investigations analyzing the role of intratumor microbes in ICI responses. A recent study demonstrated that probiotic *Lactobacillus reuteri* can translocate from the gastrointestinal tract to the TME in melanoma [120,121]. Within the tumor, *L. reuteri* metabolizes dietary tryptophan into indole-3-cabaldehyde (I3A) to promote CD8+ T cell activity in the TME and enhance the efficacy of anti-PD-L1. An analysis of tumor samples from patients with metastatic renal cell carcinoma also showed that patients who responded well to anti-PD-1 therapy had a higher relative abundance of *Corynebacterium* in contrast to non-responders [121,122]. Further relevant to mention, recent studies interestingly suggest Phosphodiesterase 5 inhibitors (PDE5Is), which are traditionally used for non-malignant reasons, may enhance anticancer immunity by modulating immune cell activity within the tumor microenvironment—underscoring their promise as repurposed agents in cancer treatment [123].

### 6.4. The Crosstalk Between Genetics, Microbiome, and Immunotherapy

Cancers, in general, are regulated either by gene expression changes or genetic alterations. These also could play a key role in the context of immunotherapy treatment outcomes alongside the body’s microbiome. The genetic composition of the host experiencing cancer, along with the bacteria that make up the microbiome—whether from the target tissue or the gut—can be significant factors. For example, it has been shown that toxins released by gut bacteria can alter the genetic makeup of tissues, which may have long-lasting effects on treatment outcomes [124,125]. In the case of colorectal cancer (CRC), it has been demonstrated that certain bacteria coexist with a specific genetic makeup of the host in both tumor and non-tumor tissues [126]. This scenario could potentially determine the strategies of treatment, especially those that are immunotherapy-based in many cancers.

### 6.5. Microbiome Variability Across Ethnic Groups

The composition of the microbiome varies in ethnic groups due to a myriad of factors, including genetics, environment, diet, and culture [127,128]. Such differences may contribute to the varied responses to ICIs and other cancer treatment options [122]. For example, patients of African descent and patients from cultures that adhere to a Mediterranean diet or diets with high levels of fiber have a very diverse microbiome, which may improve responsiveness to ICIs as patients with increased gut alpha diversity had more positive outcomes [129,130,131]. Another study has shown that mRCC patients of Latino background who received combined anti-PD-1/-anti-CTLA-4 tend to have lower progression-free survival rates compared to non-Latino patients [132]. Interestingly, an exploratory analysis of the microbiome of Latino mRCC patients demonstrated a higher Firmicutes/Bacteroidetes ratio and enrichment of *Roseburia spp.* and *Eubacterium rectale*, which are linked to favorable ICI outcomes, suggesting that further analysis is required to understand the full scope of microbial differences across ethnicities and impacts on ICI responses.

Understanding the influence of the microbiome and its metabolites offers opportunities to enhance immunotherapy outcomes for diverse populations. Tailoring probiotic interventions, prebiotic-rich diets, or FMTs based on patient microbiome profiles could improve response rates across patients of varying backgrounds and reduce AEs.

## 7. Leveraging the Microbiome for Better Immunotherapy Outcomes

Harnessing the gut microbiome has become an exciting frontier in boosting cancer immunotherapy effectiveness. This intricate ecosystem of microbes plays a vital role in shaping the immune system, including how patients respond to ICIs. Research has uncovered that certain gut bacteria can enhance antitumor immunity, directly influencing the success of these treatments. Recent insights into the gut microbiome are shaping innovative strategies to modulate its composition, employing tools such as dietary adjustments, probiotics, prebiotics, and FMT. Researchers are exploring these methods not only to boost the effectiveness of treatments but also to improve the quality of life for cancer patients by leveraging the gut’s profound influence on immune function [89,133].

### 7.1. Prebiotics, Probiotics, and Postbiotics in Cancer Immunotherapy

Prebiotics, probiotics, and postbiotics are increasingly recognized for their pivotal roles in gut health and their potential impact on cancer immunotherapy outcomes. Prebiotics, indigestible fibers that promote the growth of beneficial gut bacteria, are complemented by probiotics, which are live microorganisms that confer health benefits upon consumption. Postbiotics, the bioactive compounds produced by these microbes, directly influence host health through mechanisms such as immune modulation. Prebiotic-rich diets, particularly those high in fiber, have been linked to beneficial shifts in gut microbiota and heightened immune activity. Despite these encouraging findings, responses vary significantly across populations and cancer types, emphasizing the need for comprehensive clinical trials to clarify these relationships [134,135].

### 7.2. The Role of Fecal Microbiota Transplantation

FMT represents a novel therapeutic pathway by reintroducing microbial diversity through the transfer of stool from rigorously screened donors. By restoring balance to the gut microbiota, FMT has the potential to augment the efficacy of ICIs by modulating immune responses and influencing the TME. This approach introduces beneficial bacteria capable of stimulating immune cells and mitigating immunosuppressive signals, thereby creating a more favorable environment for antitumor activity. Addressing gut dysbiosis, often associated with poor treatment outcomes and inflammation, further highlights its therapeutic potential. Nonetheless, integrating FMT into clinical practice requires overcoming significant hurdles, including ensuring safety through stringent donor screening and the standardization of protocols to minimize risks like pathogen transmission or immune complications. Research is also needed to refine its application, from optimizing donor selection to determining effective treatment schedules. As advancements continue, FMT holds promise as a tool for precision medicine, with the potential to revolutionize cancer immunotherapy [136,137].

### 7.3. Preclinical and Clinical Innovation and Insights in Microbiome-Based Therapies

Innovations in microbiome research are opening doors to enhanced cancer immunotherapy through the integration of approaches like prebiotics, probiotics, and FMT. Personalizing these interventions, such as through microbiome profiling, allows for treatments tailored to individual patient needs, improving efficacy while reducing side effects. Exciting developments include engineered probiotics capable of delivering therapeutic agents directly to tumors, minimizing systemic toxicity. Synthetic postbiotics, designed to modulate immune responses, also show potential in improving treatment outcomes. The adoption of multi-omics technologies, which integrate genomics, proteomics, and metabolomics, is transforming patient profiling by enabling the precise identification of biomarkers and targeted therapies. Together, these advancements exemplify a synergistic approach combining cutting-edge science with precision medicine to elevate cancer care [138].

There is continued resistance to standard-of-care treatments in cases of metastatic and locally advanced cancer, metastatic melanoma, renal cell carcinoma, pancreatic carcinoma, and lung cancers such as non-small cell lung carcinoma and mesothelioma. This has presented a clear need for research into potential modulators of this tumor immunity to improve treatment outcomes and, thus, patient survival [3]. Research into the gut microbiota has already demonstrated that it plays a vital role in maintaining host health [139] and that changes to its composition can have strong negative health effects on the host body, including influencing the development and progression of various cancers [6,122,138]. Research focusing on ICI therapy in mouse models has already shown that specific species of bacteria within the gut microbiota, *A. muciniphila* and *Bifidobacterium*, can modulate cancer treatment outcomes [89,111]. Additionally, in preclinical studies, researchers have demonstrated that alterations to the gut microbiome can influence the immune system’s response to common chemotherapeutic drugs like cyclophosphamide [140]. With preclinical insights into the relationship between the gut microbiota and cancer immunotherapy outcomes demonstrated, the groundwork for clinical investigations has been laid and focus has shifted to the potential real-world application of this discovery. From this, fecal microbiota transplants have emerged as a promising strategy to enhance cancer treatment outcomes and reduce treatment toxicities to the patient by modulating the gut microbiome.

FMT is the most direct method available to alter the gut microbiome, it involves transferring stools from a healthy donor to a recipient through oral capsules or colonoscopy with the aim of restoring a beneficial microbiota profile or altering the microbiota towards a goal composition. FMT is already commonly used in the treatment of *Clostridiodies difficile* infections to rebuild the gut microbiota to support recovery from the infection. A clinical trial [137] based on this previous research [122,141] investigated the safety and efficacy of treating anti-PD-1 refractory metastatic melanoma with the reinduction of ICI therapy in combination with FMT in a cohort of 10 patients. Within the cohort it was found that three of the 10 patients had new responses to treatment with positive changes in the composition of immune cell infiltrates in both the intestinal environment and the TME, including increased CD8+ T cell activation and a decrease in IL-8 expressing cells, signifying a better immune response to the tumor. Building off this research, there is a variety of in-preparation and ongoing Phase I and II clinical trials reported in the FDA database investigating FMT with immunotherapy as a combination treatment for resistant tumors like those of melanoma described above. As this is quite a novel field of study, many of the clinical trials are currently focused on patient safety with hopeful progression to the investigation of intervention effectiveness in the coming years (Table 1).

## 8. Conclusions and Future Directions

The influence of the microbiome on cancer physiology, treatment, and prognosis is becoming well-recognized. Factors such as community structure, pathogens, and microbial metabolites interact with host cells through various mechanisms to shape the TME and modulate responses to classical and novel treatment modalities. The altered metabolism within the TME provides a niche for the proliferation of microbes that maintains a dynamic equilibrium with the host tissue and with the intestinal microbiome.

As ICI therapy continues to show promise as a therapeutic option, the interactions of this technology with the microbiome need to be investigated. The influence of gut and intratumor microbiota on ICI therapies encompass numerous mechanisms such as modulating inflammatory response through SCFAs, enhancing anti-cancer T cell activity by acting as an adjuvant in blockade therapy and decreasing treatment efficacy through the production of virulence factors, and the proliferation of pathogenic species. In particular, future research could explore the impact of the microbiome on immune cell activation and its potential to inhibit immune checkpoint pathways. Additionally, investigating the role of the microbiome in modifying the tumor microenvironment (TME) could further reveal its influence on therapeutic responses. Beyond bacteria, the role of viruses, fungi, and protozoa that inhabit the host is arising as an important factor in their potential influence on treatment outcomes. This growing understanding highlights the need for further exploration of how these microorganisms collectively shape the TME and immune responses. Interactions continue to be investigated; an important aspect of the microbiome that comes to light is the contribution of not just the bacteria but also the viruses, fungi, and protozoa that inhabit the host and their potential influence on treatment outcomes.

Several avenues in this field are currently under investigation to harness the power of the microbiome to improve treatment outcomes and efficacy. Technologies such as FMT and supplementation with pre- or probiotics have demonstrated promising potential in the clinical setting to improve patient outcomes. Additionally, studies that demonstrate the interplay between the gut microbiota and other host microbiomes, such as the skin, oral, and lung microbiome, may reveal concerted relationships that influence treatment efficacy. By leveraging the host microbiome with current oncology, there is potential to not only improve treatment outcomes but also minimize adverse effects to enhance patient care.

## Figures and Tables

**Table 1:** Clinical trials investigating the interaction of the microbiome with ICI therapy.

NCT Number	Title	Status	Conditions	Aims	Sponsor	Phase
NCT06050733	The Gut Microbiome and Immunotherapy Response in Solid Cancers	Recruiting	Malignant nonresectable or metastatic solid cancer	Characterize gut microbiome of solid cancer patients that have disease progression during standard of care treatment with PD-L1 blockade. Assess measures of cognition and fatigue in solid cancer patients undergoing standard of care treatment with PD-L1 blockade and associate with composition of gut microbiome.	University of Texas Medical Branch, Galveston, Texas, United States	Phase I
NCT05251389	FMT to Convert Response to Immunotherapy	Recruiting	Advanced stage cutaneous melanoma (III or IV) requiring treatment with PD-L1 blockade	To assess whether the transfer of gut microbiota can convert response to immunotherapy checkpoint inhibitors in refractory metastatic melanoma patients.	The Netherlands Cancer Institute, Amsterdam, Netherlands	Phase Ib/II
NCT04163289	Preventing Toxicity in Renal Cancer Patients Treated with Immunotherapy Using Fecal Microbiota Transplantation	Active, not recruiting	Advanced, nonresectable or metastatic renal cell carcinoma (RCC)	Investigate the safety and efficacy of fetal microbiota transplantation as a supportive therapy to immunotherapeutic for RCC such as ipilimumab with the goal of reducing immune related toxicities in patients.	Lawson Health Research Institute, Ontario, Canada	Phase I
NCT06448572	EXL01 in Combination with Nivolumab for Advanced NSCLC Refractory to Immunotherapy.	Recruiting	Non-small cell lung cancer (NSCLC)	Investigate the efficacy of a combination NSCLC treatment using nivolumab and EXL01, a strain of F. prausnitzii, a member of the normal gut microbiota based on known association of the strain with antineoplastic immunotherapy.	University Hospital, Lille, France	Phase I/II
NCT05354102	A First-in-human Combination Treatment Study with a Single Dose Level of BMC128	Active, not recruiting	Non-small cell lung cancer (NSCLC), melanoma, and renal cell carcinoma (RCC)	To assess safety, patient tolerability and effects on the cancer treatment and gut microbiome using nivolumab in a combination treatment with BMC128, a combination of four naturally occurring commensal intestinal bacterial strains.	Biomica Ltd., Haifa, Israel	Phase I
NCT04645680	Diet and Immune Effects Trial: DIET- A Randomized Double Blinded Dietary Intervention Study in Patients With Metastatic Melanoma Receiving Immunotherapy	Active, not recruiting	Stage III and IV cutaneous and metastatic melanoma	To investigate the effects of dietary intervention on the structure and function of gut microbiota as a modulator of systemic and tumor immunity using two different diets in patients being treated with standard of care pembrolizumab or nivolizumab.	M.D. Anderson Cancer Center, Houston, Texas, United States	Phase II
NCT06403111	FMT + Immunotherapy + Chemotherapy as First-line Treatment for Driver-gene Negative Advanced NSCLC	Not yet recruiting	Inoperable, treatment resistant locally advanced or metastatic non-small cell lung cancer	To investigate the safety and treatment response of receiving FMT combined with tislelizumab + pemetrexed and platinum-based therapy (adenocarcinoma patients)/albumin-bound paclitaxel and platinum-based therapy (squamous cell carcinoma patients).	Changzhou People’s Hospital, China	Phase II
NCT06551272	Microbiota Modification for Immuno-oncology in Hepatocellular Carcinoma	Not yet recruiting	Locally advanced nonresectable or metastatic hepatocellular carcinoma	To investigate the concept of using EXL01, a pharmacological preparation of F. prausnitzii in combination with atezolizumab-bevacizumab for Hepatocellular carcinoma patients based on previous research using EXL01 in combination with immunotherapy for melanoma.	Center Eugene Marquis, Rennes, France	Phase II
NCT05462496	Modulation of the Gut Microbiome with Pembrolizumab Following Chemotherapy in Resectable Pancreatic Cancer	Recruiting	PDAC	To determine the change in immune activation in pancreatic tumor tissue following treatment with antibiotics and pembrolizumab. To further investigate changes in the microbiome as measured in tumor and stool following treatment to correlate treatment response to microbiome changes.	Icahn School of Medicine at Mount Sinai, New York, New York, United States	Phase II
NCT05502913	Fecal Microbiota Transplantation to Improve Efficacy of Immune Checkpoint Inhibitors in Metastatic Lung Cancer	Recruiting	Metastatic lung cancer treated with immune checkpoint inhibitors	Evaluate the safety and efficacy of fecal microbiota transplantation in altering response to immunotherapy in patients with metastatic lung cancer, to determine possible benefits of future FMT-based combination therapy.	Soroka University Medical Center, Beer Sheva, Israel	Phase II
NCT05286294	Microbiota Transplant to Cancer Patients Who Have Failed Immunotherapy Using Feces from Clinical Responders	Recruiting	Metastatic melanoma, head and neck squamous cell carcinoma, NSCLC, and clear cell RCC	Evaluate the efficacy of fecal microbiota transplant from treatment-responsive patients to cancer patients not responding to immunotherapeutics such as PD-L1 checkpoint inhibitors.	Oslo University Hospital, Oslo, Norway	Phase II
NCT05865730	A Phase 1/2 Study of Oncobax^®^-AK Administered in Combination with Immunotherapy to Patients with Advanced Solid Tumors	Recruiting	Renal cell carcinoma and non-small cell lung carcinoma	To analyze the gut microbiota in NSCLC and RCC patients that harbor the Akkermansia bacteria and assess its effects on reversing resistance to PD-1 blockade demonstrated in preclinical models.	EverImmune, Dijon, France Institut Gustave Roussy, Paris, France	Phase II
NCT05750030	FMT Combined with Atezolizumab Plus Bevacizumab in Patients with Hepatocellular Carcinoma Who Failed to Respond to Prior Immunotherapy	Recruiting	Hepatocellular carcinoma refractory to atezolizumab/bevacizumab	Assess the effects of FMT in combination with atezolizumab plus bevacizumab on treatment response and patient quality of life. Additionally, to explore the effect of FMT on recipient gut microbiota composition and immune activity in the gut microenvironment.	Medical University of Vienna, Vienna, Austria	Phase II
NCT03891979	Gut Microbiome Modulation to Enable Efficacy of Checkpoint-based Immunotherapy in Pancreatic Adenocarcinoma	Withdrawn	Pancreatic ductal adenocarcinoma	Phase IV study to investigate the effects of intestinal microbiota modulation using antibiotics in conjunction with pembrolizumab for treatment of surgically resectable pancreatic cancer.	NYU Langone Health, New York, New York, United States	Phase IV
			Observational Studies			
NCT04954885	Evaluating Biomarkers for the Prediction of Immunotherapy Response and Toxicity	Recruiting	Stage III and stage IV non-squamous non-small cell lung cancer	To investigate the performance of the gut microbiome and functional status as predictive biomarkers of clinical benefit for lung cancer patients receiving randomized treatment combinations.	The Ohio State University Comprehensive Cancer Center, Columbus, Ohio, US	
NCT06318507	The Intestinal Microbiome in Triple Negative Breast Cancer Treated With Immunotherapy	Recruiting	Triple-negative breast cancer patients with BMI greater than or equal to 25 on anti-PD-1 therapy	To investigate how the intestinal microbiome changes between patients with triple negative breast cancer and obesity who respond to anti-PD-1 therapy and those who are non-responders.	Pennington Biomedical Research Center, Baton Rouge, Louisiana, US	
NCT04957511	Feasibility Study of the Interplay Between the Host Gut Microbiome and Efficacy of Treatment for Advanced or Recurrent Gynecological Cancer Patients Receiving Immunotherapy	Recruiting	Advanced or recurrent gynecological cancer patients receiving immunotherapy	To investigate the gut microbiome and changes in composition of said microbiome in patients undergoing immunotherapy for advanced or recurrent gynecological cancer.	Advent Health Cancer Institute, Orlando, Florida, US	
NCT04291755	Development and Analysis of a Sample Bank for Cancer Patients, Enabling the Systematic Study of the Effect of Blood, Urinary Tract, and Gut Microbiomes on Response	Recruiting	Patients undergoing any form of cancer immunotherapy	To obtain samples of blood, urine, and stool from subjects with cancer under immunotherapy treatment to establish a sample bank for further research into gut microbiota populations in cancer treatment.	Compassionate Care Research Group Inc., Fountain Vally, California, US	
NCT04054908	Gut Microbiome in Colorectal Cancer	Completed	Colorectal cancer	To investigate the alterations in the gut microbiome of three cohorts of patients receiving treatment for colorectal cancer including chemotherapy, radiotherapy, and immunotherapy.	University of California San Francisco, San Francisco, California, US	

## Abbreviations

	Definitions
APC	Antigen-presenting cells
CAF	Cancer-associated fibroblasts (CAFs)
CD	Cluster of Differentiation
CEACAM	Carcinoembryonic antigen-related cell adhesion molecules
CRC	Colorectal cancer
CTLA-4	Cytotoxic T-lymphocyte associated protein-4
CTX	Chemotherapy
dMMR	Deficient mismatch repair
DCA	Deoxycholic acid
DC	Dendritic cells
EBV	Epstein Barr virus
ECM	Extracellular matrix
FMT	Fecal microbiota transplantation
5-FU	5-fluorouracil
GC	Gastric cancer
HBV	Hepatitis B virus
HCV	Hepatitis C virus
HIF	Hypoxia-inducible factor
HPV	Human papillomavirus
HTLV-1	Human T-cell lymphotropic virus type 1
ICIs	Immune checkpoint inhibitors
irAEs	Immune-related adverse events
ITIM	Immunoreceptor tyrosine-based inhibitory motifs
LAG-3	Lymphocyte activation gene 3
LPS	Lipopolysaccharide
MDSCs	Myeloid-derived suppressor cells
MSI-H	Microsatellite instability-high
NK	Natural killer
PDAC	Pancreatic ductal adenocarcinoma
PD-1	Programmed cell death protein-1
PD-L1	Programmed cell death protein-1 ligand
PRR	Pattern recognition receptors
ROS	Reactive oxygen species
RT	Radiation therapy
SCFA	Short-chain fatty acids
TAMs	Tumor-associated macrophages
TIM-3	T-cell immunoglobulin and mucin domain 3
TIME	Tumor-immune microenvironment
TIGIT	T-cell immunoglobulin and ITIM
TIL	Tumor-infiltrating lymphocytes
TME	Tumor microenvironment
Tregs	Regulatory T cells
